# New insights into the role of age and carcinoembryonic antigen in the prognosis of colorectal cancer

**DOI:** 10.1038/sj.bjc.6604114

**Published:** 2007-11-20

**Authors:** P G Gobbi, F Valentino, E Berardi, C Tronconi, S Brugnatelli, O Luinetti, R Moratti, G R Corazza

**Affiliations:** 1 Sezione di Clinica Medica I (Dipartimento di Medicina Interna), Università di Pavia, Fondazione IRCCS Policlinico S Matteo, Pavia, Italy; 2 Sezione di Anatomia Patologica (Dipartimento di Patologia Umana ed Ereditaria), Università di Pavia, Fondazione IRCCS Policlinico S Matteo, Pavia, Italy; 3 Servizio di Analisi Chimico-Cliniche, Università di Pavia, Fondazione IRCCS Policlinico S Matteo, Pavia, Italy

**Keywords:** colorectal cancer, age, CEA, staging, relative survival

## Abstract

The aim of this study was to verify through relative survival (an estimate of cancer-specific survival) the true prognostic factors of colorectal cancer. The study involved 506 patients who underwent locally radical resection. All the clinical, histological and laboratory parameters were prognostically analysed for both overall and relative survival. This latter was calculated from the expected survival of the general population with identical age, sex and calendar years of observation. Univariate and multivariate analyses were applied to the proportional hazards model. Liver metastases, age, lymph node involvement and depth of bowel wall involvement were independent prognosticators of both overall and relative survival, whereas carcinoembryonic antigen (CEA) was predictive only of relative survival. Increasing age was unfavourably related to overall survival, but mildly protective with regard to relative survival. Three out of the five prognostic factors identified are the cornerstones of the current staging systems, and were confirmed as adequate by the analysis of relative survival. The results regarding age explain the conflicting findings so far obtained from studies considering overall survival only and advise against the adoption of absolute age limits in therapeutic protocols. Moreover, the prechemotherapy CEA level showed a high clinical value.

A large body of investigational data demonstrates that the prognosis of patients undergoing bowel resection for colorectal cancer is mainly determined by factors related to local tumour growth and the presence or absence of nodal and/or distant metastases. Many classification systems have been devised to categorise these anatomical factors for clinical use but an increasing number of new pathological and nonanatomical elements show interesting correlations with survival and would be worth testing systematically for selective integration into the available staging classifications. Improvement of the prognostic accuracy of these classifications might allow a more flexible use of the increasing number of new drugs and therapeutic options now available for postsurgical management of patients with colorectal cancer. However, since this malignancy is a disease of the elderly and the populations of developed countries are ageing rapidly, overall survival, as currently investigated, may not be the most suitable outcome parameter for evaluating the real prognostic impact of tumour-linked factors. Because about one-half of all colorectal carcinomas in our series occur in people aged 65 years or older, and a considerable number of these subjects die of other causes with no evidence of cancer, a fraction of these deaths should not be related to the tumour.

There are two main purposes of the present work: (1) to verify the prognostic significance of the current clinicopathological factors through a study of both overall and relative survival, this latter being a selective estimate of the chance of surviving the effects of cancer; (2) to include – among the factors to be tested – the linear dimensions of the resected tumour as an estimate of its preoperative volume. Tumour size has never shown a clear prognostic value (Newland *et al*, 1994; Frank *et al*, 1995; Takahashi *et al*, 1997), but its potential effect has always been evaluated through overall survival analyses, in which it has shown a satisfying correlation with carcinoembryonic antigen (CEA) and carbohydrate antigen 19-9 (CA19-9) levels (Louhimo *et al*, 2002; Yuste *et al*, 2003). Since these markers are commonly considered of prognostic value (Kanellos *et al*, 2006a), but are inconstantly expressed, a re-evaluation of the predictive value of tumour size through an analysis of relative survival might yield definitive results on this matter.

## PATIENTS AND METHODS

### Patients

From 1 January 1994 to 31 December 2002, 536 patients were referred to the Day Hospital of the Clinica Medica I after local radical resection of colonic carcinoma (*n*=324) or rectal carcinoma (*n*=182). These two groups of patients were studied together since their survival proved to be very similar. During the time of the study, there was not active clinical research on colorectal cancer in the Clinica Medica I, and patients were referred to the Day Hospital from the surgical divisions of the San Matteo Hospital and from the hospitals in the neighbourhood of 20–30 km. The following information was collected for each patient presenting signs and symptoms, location of the tumour, description of the surgical operation, radicality of the resection performed, macroscopic features at presentation, diameters of the tumour mass, number of regional metastatic lymph nodes, contiguous viscera involved, number and diameters of distant metastatic lesions, microscopic subtype of the tumour, depth of penetration into the bowel wall, cell differentiation, grade of lymphatic, venous and perineural invasion, metastatisation of the collected lymph nodes and main laboratory data at the start of adjuvant chemotherapy and about 5–6 weeks after surgery (blood cell count, serum protein electrophoresis, liver and kidney function tests, and serum levels of CEA and CA19-9).

Macroscopic evaluation of the whole resected material and histological examination of the sampled specimens were performed centrally. Vascular and lymphatic invasions were evaluated on paraffin sections stained with haematoxylin–eosin; cases in which recognition of endothelial structures was uncertain underwent immunohistochemical search for CD34 and CD31 markers. In fact, both the anti-CD31 antibody, which identifies the antigen ER-MP12, identical to the vascular endothelial adhesion molecule PECAM-1, and the anti-CD34 antibody, which stains normal and endothelial cells, make the identification of vascular and lymphatic vessels easier. Neural invasion was always evaluated through haematoxylin–eosin staining.

Carcinoembryonic antigen and CA19-9 were measured after surgery, before the start of chemotherapy (if any) by two sites, noncompetitive immune assays performed on an automated immunochemistry analyzer with chemiluminescence detection (Advia Centaur, Bayer Diagnostics, Tarrytown, NY, USA). The measurement ranges for CEA and CA19-9 were 0.5–100 ng ml^−1^ and 1.2–700 U ml^−1^, respectively; when results exceeded the upper limit of the analytic range, serum was diluted according to the manufacturer's instructions. Quality control was ensured by assaying three levels of control sera in each analytical series within a 3-monthly European interlaboratory control programme. Several patients in the present series were referred postoperatively to our unit from a neighbouring hospital, often with preoperative CEA measurements that were not technically comparable or had not even been assessed.

For the purposes of this study, patients alive in 2005 who had not had a medical examination within the preceding 6 months were recalled for a new clinical and instrumental control. The vital status of those patients who did not respond to this recall was ascertained by telephone or investigated in the General Registry Offices of their last known municipality of residence. Thirty patients were excluded from the study because of incomplete data regarding either surgical resection or pathological findings. Thus, 506 patients formed the population of the study. All were staged according to Dukes' classification (Dukes, 1940), the modified Astler–Coller classification (Astler and Coller, 1954) and the TNM classification (American Joint Committee on Cancer, 2002). The main characteristics of the study population are reported in Table 1.

Unless there were particular clinical conditions, adjuvant chemotherapy was administered according to the following criteria: stages II and III patients were treated with the regimen proposed by Machover *et al* (1982) (5-fluorouracil plus folinic acid, at the doses of 370 and 100 mg m^−2^, respectively, with daily i.v. bolus injection for 5 days every 28 days, for six cycles). Until 1998, patients in stage IV were treated with the schedule described by De Gramont *et al* (1997) (5-fluorouracil 400 mg m^−2^ in i.v. bolus injection and 600 mg m^−2^ in continuous infusion plus folinic acid 100 mg m^−2^ in a 2-h infusion for 2 days every 14 days for 12 times), in six cases also combined with regional intra-arterial 5-fluorouracil infusion for liver metastases. After 1998, patients in stage IV were administered either infusional fluorouracil or FOLFIRI (Andre *et al*, 1999) or FOLFOX 4 regimens (De Gramont *et al*, 2000) (these last are De Gramont-like schedules with the addition of either irinotecan 180 mg m^−2^ or oxaliplatin 85 mg m^−2^ on the first day, respectively). Since 2000, 11 patients with liver metastases were spared locoregional chemotherapy and underwent radiofrequency thermoablation. Forty-one patients with rectal carcinoma also received local radiotherapy. Survival of the patients treated in the last 4 years of the study, when analysed stage by stage, tended to be better than that of the first quadrennium, but differences were not statistically significant.

### Statistics

The time parameters taken into account were overall survival and relative survival. This latter was calculated as the ratio of the overall survival rate observed in the patient population and the expected survival rate drawn from the general reference population for subjects similar to the patients with respect to age, sex, calendar year of initial observation and length of observation (Armitage and Berry, 1987). The age-, gender-, and calendar year-specific death rates available from the National Italian Mortality Tables (ISTAT, Istituto Nazionale di Statistica) were used to calculate the expected deaths – and so the expected survival. The age changes according to individual birthdays in every year of the follow-up were taken into account. In this way, each patient was considered to have a wide control group from the general population with corresponding anagraphic characteristics with a well-defined probability of dying (or surviving). Consequently, the relative survival, obtained by adjusting observed survival for normal life expectancy, can be considered a satisfactory estimate of the chance of surviving the effects of cancer. In detail, and for example, the expected probability of death (from mortality tables) of a man born on 1 August 1926, who survived the whole 1997 is that of a 70-year-old man during the first 212 days of the year (0.03063 per 100 000) and that of a 71-year-old man in the remaining 153 days (0.03376 per 100 000): the resulting probability of death, expected from the reference population and to which the subject was exposed during the whole 1997, will be 0.03063 × 212/365+0.03376 × 153/365=0.03194. The probability of death of 1 year must be added to that of any other year (or fraction of year) of the follow-up. The observed deaths recorded in the patient population at the end of the follow-up time and the cumulative expected probability of death during the corresponding time obtained from the mortality tables of the general population are the variables that can be used in both survival calculations and multivariate analyses.

The Kaplan–Meier method (Kaplan and Meier, 1958) was used to evaluate survival, and differences were analysed by the Log-rank test (Peto *et al*, 1977). The clinical and pathological features that showed statistically significant prognostic value in univariate analyses were selected for multivariate analyses. These were performed by multiple regressions applied to a Cox proportional hazards model (Cox, 1972). A stepwise selection of factors was applied to the multiple regressions.

## RESULTS

**Figure 1:**
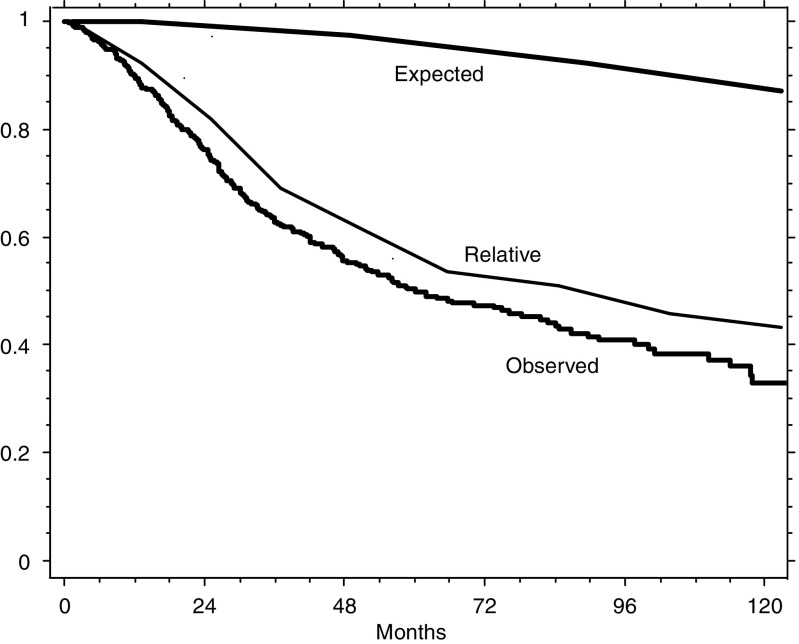
Overall, expected and relative survivals of the 506 patients with colorectal cancer. Expected survival was that of comparable subjects of the general reference population, and relative survival was calculated from the overall and the expected survivals (see Patients and Methods).

The median length of the follow-up of all patients was 54.6 months (62.8 for those alive). The range was from 3 to 144 months. Figure 1 illustrates the survival observed in our series of patients, the survival expected in a corresponding general reference population and the relative survival of our patients, computed from the data of the first two curves. The difference between the observed and relative survivals is due to the approximately 12% of deaths expected to occur from causes other than colorectal cancer (observed/expected deaths: 217/27). Since the number and distribution along time of these expected deaths may be a confounding factor in the identification of truly prognostic determinants, we verified against the relative survival the results obtained for overall survival in both the univariate and multivariate analyses.

Table 2 shows the results of the univariate evaluation of the prognostic value of all the clinical and pathological factors considered in relation to both the survival parameters. Most of the factors that are significantly related to overall survival are also related to relative survival, though with considerable differences. The analysis against relative survival seems to reveal an individual role – not emerging from the study of overall survival – for sex and neuroinvasion. Tumour size shows no prognostic value with regards to either survival parameter. The staging systems have the highest correlation with both overall and relative survival without a clear prevalence for one system over the others.

All the single factors (i.e., excluding the staging systems) that demonstrated a significant prognostic value at univariate analysis were entered into the multivariate evaluation. The final results of the stepwise selection of variables are reported in Table 3, all the other factors having been excluded step by step as not contributing significantly to the model. Three out of the five most powerful prognostic determinants are the main individual parameters, which are incorporated in the current staging classifications (depth of bowel wall involvement, number of regional lymph nodes involved and presence of liver metastases). The coefficient of the fourth factor, age, has an opposite sign according to whether overall or relative survival is considered. Indeed, age is directly correlated with overall survival and inversely with relative survival. The level of CEA is better related to survival than is the level of CA19-9, although it retains a clearly prognostic role only for relative survival (while it approaches statistical significance for overall survival).

**Figure 2:**
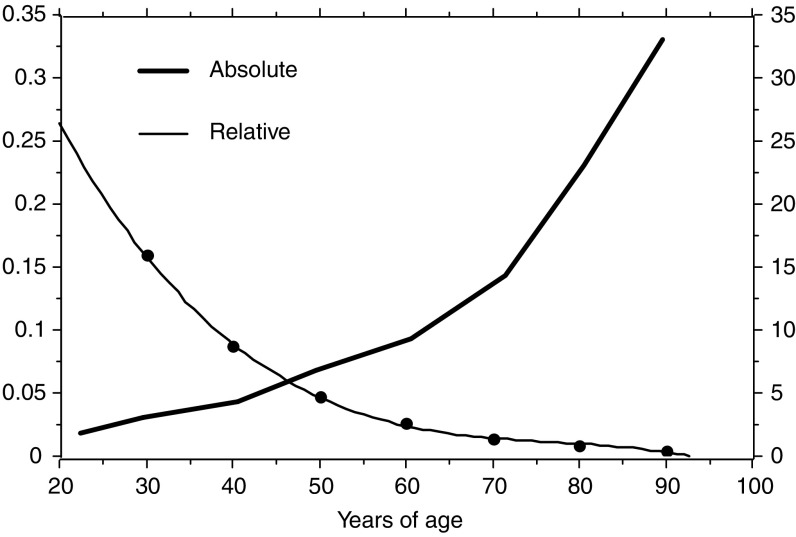
Different curves of the hazard rate estimated from the coefficients of the final step of the multivariate analysis in relation to either the overall (absolute) or the relative (specific) survival. The curve of the overall survival (thick line) refers to the vertical axis on the right, that of the relative survival (thin line) refers to the ordinate on the left. The graph reports the hazards related to the age obtained from multivariate analysis, (i.e., after consideration of the other factors important for survival – they are not crude curves of observed hazards). The opposite scales of the two vertical axes indicate the very different entity of the variation of the hazard with age, the different slopes of the curve show the opposite role of the age when multivariately evaluated against overall (absolute) or relative (specific) survival.

Figure 2 illustrates the two different curves of the hazard rate by age drawn from the coefficients of the multivariate analysis related to either overall or relative survival. Both these curves are an expression of the individual role of age when computed multivariately, that is, after consideration of the other factors important for survival (they are not crude curves of observed hazards). It is clear that the role exerted by age on relative survival is much weaker – although still statistically significant – than that on overall survival, but shows a clear trend to decrease in the elderly, in contrast with the marked increase with respect to overall survival.

**Figure 3:**
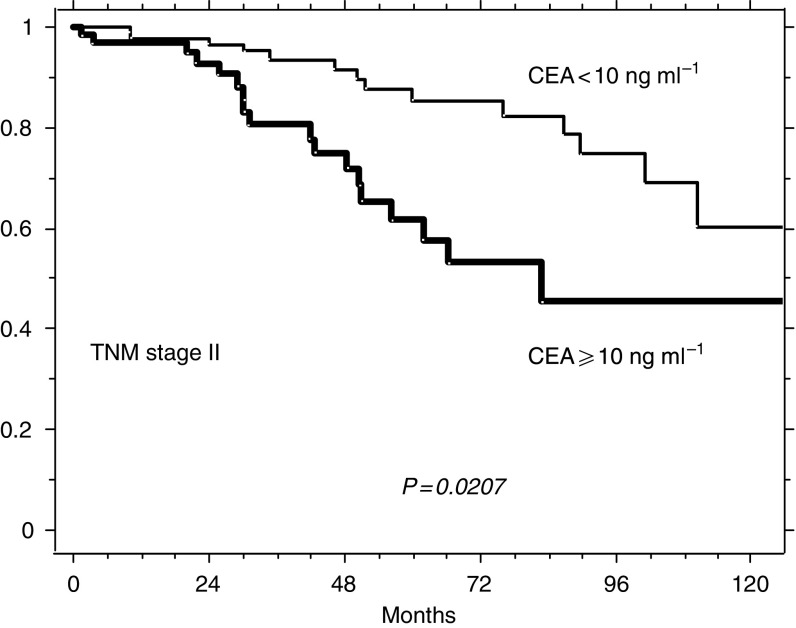
Relative survival of 189 patients with TNM stage II (A+B) according to whether their prechemotherapy levels of CEA were ⩾ or <10 ng ml^−1^ (65 and 124, respectively).

**Figure 4:**
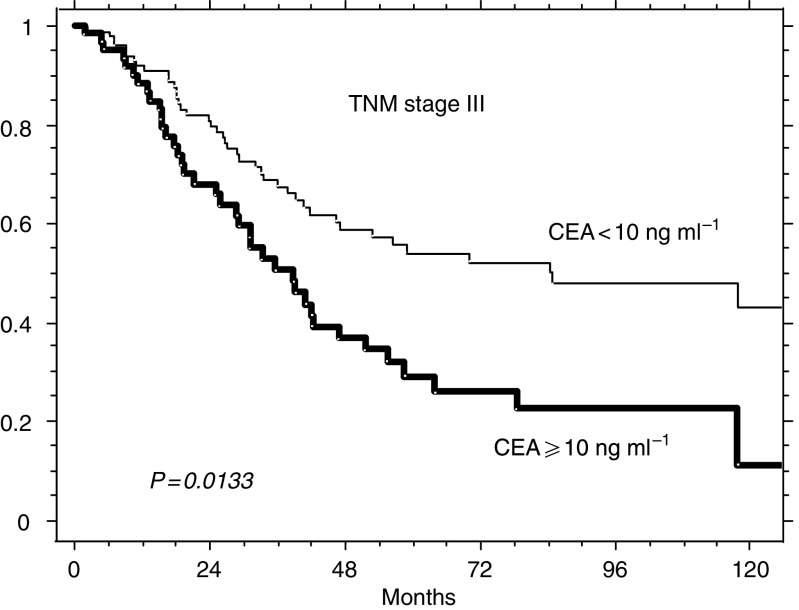
Relative survival of 176 patients with TNM stage III (A+B+C) according to whether their prechemotherapy levels of CEA ⩾ or <10 ng ml^−1^ (64 and 112, respectively).

**Figure 5:**
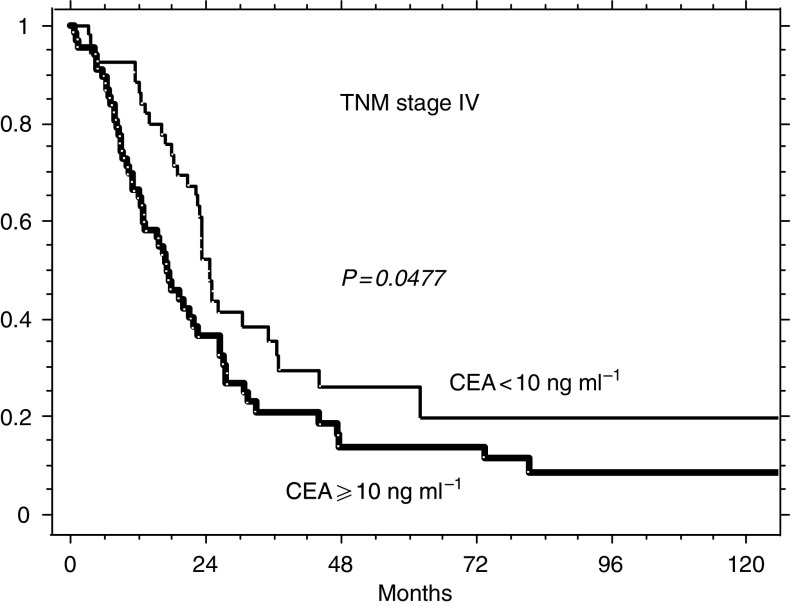
Relative survival of 125 patients with TNM stage IV according to whether their prechemotherapy levels of CEA ⩾ or <10 ng ml^−1^ (39 and 66, respectively).

Figure 3 , 4 and 5 illustrate the possible integration of CEA levels into one of the current staging systems (TNM). The survival of the 189 patients of this series presenting with TNM stage II (A+B), of the 176 with stage III (A+B+C) and of the 125 stage IV patients can be further split according to whether prechemotherapy levels of CEA were ⩾ or <10 ng ml^−1^. The choice of testing the concentration of 10 ng ml^−1^ as a potential prognostic discriminant was made for mere illustrative purposes (the analysis of Table 3 does not indicate any distinct threshold level as most suitable for clinical use, but suggests that the prognostic value of postoperative CEA is more probably related to the whole distribution of its levels).

## DISCUSSION

Checking prognostic factors evaluated in relation to overall survival for their significance to relative survival is a way of recognising and separating what of the patients' fate depends strictly on cancer and what depends on the large number of comorbid conditions that increasingly affect the elderly. Since the age of the population is increasing and, moreover, the incidence of colorectal cancer rises with age, it is useful to adjust the overall survival of these cancer patients according to the expected mortality from all causes of death. The datum used for this purpose is the expected mortality in the general population with exactly the same age, sex and length of observation as for the group of patients. Note that by this method, sex and age are considered prognostic factors already present in the general population, as they undoubtedly are, and can be considered prognostic factors of the disease under investigation only if the role usually exerted on the general population is significantly altered. The relative survival obtained in this way is a very good estimate of the specific survival, moreover, achieved without the well-known difficulty of defining the exact causes of death in retrospective series. This investigation was limited to patients who underwent local radical resection of a colorectal tumour and all the data analysed were collected before the start of adjuvant chemotherapy (or follow-up, if no therapy had to be administered).

The study yielded three main results. First, the current staging systems were confirmed to be best prognosticators in colorectal cancer, since three out of the five best predictors identified by multivariate analysis are included in the criteria of the available staging systems (see Table 3, depth of intestinal wall invasion, number of regional lymph nodes involved and presence or absence of liver metastases – as the most frequent type of distant diffusion). The superiority of the staging models over any other individual factor is also evident from the comparative evaluation of the *χ*^2^-values of the univariate analysis reported in Table 2. Thus, the pivotal prognostic role of current staging systems in colorectal cancer remains undisputed after computation of relative survival.

Second, the true impact of age *per se* on the chance of surviving the direct effects of colorectal cancer has been clarified. When overall survival of patients with colorectal cancer is considered, age has the same strong and unfavourable prognostic significance as observed in most neoplastic diseases, being greater as age increases. In contrast, when relative survival is considered, age shows a weak, but statistically significant, favourable effect – a sort of mild protection. In other words, the number of unexpected deaths (i.e., those due to cancer) that can be multivariately ascribed to age, decreases slowly, but significantly, as age increases. When studying a cancer of the elderly, if analyses are restricted to overall survival, a considerable amount of mortality from other causes (e.g., infections, cardiovascular diseases, hypertension and diabetes) will be wrongly attributed to the tumour. Thus, different age ranges of the populations studied, or different age groups chosen for the analyses, together with a variable prevalence of nonneoplastic diseases in the evaluated series, can explain the discordant results in the scientific literature regarding age and colorectal cancer. Indeed, different authors have found an independent unfavourable effect of increasing age (Korenaga *et al*, 1991; Gasser *et al*, 1992; Crocetti *et al*, 1996; D'Eredita *et al*, 1996; Wolters *et al*, 1996; Payne and Meyer, 1997; Tominaga *et al*, 1997; Heys *et al*, 1998; Lagautriere *et al*, 1998; Fietkau *et al*, 2004; Munemoto *et al*, 2004), of the youngest and oldest age ranges, indifferently (Chung *et al*, 1998; Cerottini *et al*, 1999; Massacesi *et al*, 2002) or even of young age (Cai *et al*, 2005), while other investigators were not able to demonstrate any prognostic effect at all (Ponz de Leon *et al*, 1992; Wang *et al*, 2000; Mitry *et al*, 2004; Latkauskas *et al*, 2005). Only Janssen-Heijnen *et al* (2005) evaluated relative survival of patients with several cancers, utilising data from the Southern Netherlands Cancer Registry. They found that the 5-year relative survival of patients with colon cancer was slightly better in subjects ⩾70 years of age than in those <70 years old, whereas it was not affected by age in patients with indolent non-Hodgkin's lymphomas and prostate cancer, and was clearly lower in older patients with other cancers. The prevalence of comorbidity, which is claimed as a reason for less aggressive treatment in the elderly, can explain the poorer survival of older patients with most types of cancer, but seems to be inadequate for those with colon cancer. Indeed, why and how age exerts a mild protective effect on specific mortality of colorectal cancer is not clear. According to the most probable hypothesis, the tumour might progress more slowly in older patients. This idea is popular but is still debated and so far unproven. The results presented here offer indirect support for this hypothesis, but not evidence. Some genetic alterations, such as that of the promoter of the *MDM2* oncogene, are able to modify the age of onset of colorectal cancer and probably differentiate prognosis (Menin *et al*, 2006). Alternatively, adjuvant therapies may be more effective in the elderly, although this seems a rather untenable hypothesis. Certainly, age should no longer be considered the only, direct criterion for evaluating the indication of postsurgical therapies and for choosing the type of chemotherapy. Besides age, more attention should be paid to the presence of comorbid conditions, chronic diseases and functional disabilities that are frequent causes of complications and death in the elderly. A number of questionnaires have been devised with the purpose of selecting frail subjects in older cohorts (Repetto *et al*, 2001; Matthes *et al*, 2004) and probably a multiparameter evaluation should replace age alone in the selection of candidates for chemotherapy.

The third main result of this study is the independent predictive value of postsurgery CEA levels. In our multivariate analysis the postoperative level of CEA replaced that of postoperative CA19-9 as the major determinant (with a statistically relevant weight for relative survival), although the two had apparently similar prognostic roles at univariate analysis. In most of the previous studies aimed at evaluating the implications of CEA levels for staging and prognosis, the serum concentration of CEA was evaluated preoperatively and, despite some conflicting results, the majority of them showed a direct prognostic value (a nice review on this topic is available in a recent paper by Chen *et al*, 2005). Since the CEA level seems to roughly reflect the tumour burden and/or diffusion (Wanebo *et al*, 1978), its preoperative evaluation might offer a crude estimate of neoplastic spread and, thus, of the probable difficulty of achieving successful radical resection. A drop in CEA levels after the resection is considered a favourable indicator of the completeness of the surgical excision (Herrera *et al*, 1976), although CEA concentration is generally regarded as more sensitive for hepatic and retroperitoneal metastases than for local recurrence or peritoneal and pulmonary metastases (Crawford *et al*, 2003). According to Kanellos *et al* (2006c), the measurement of the CEA level in the blood intraoperatively taken from the mesenteric vein offers some advantage, as both indicator of hepatic metastases and predictor of 5-year survival. The same investigators also found a higher recurrence rate in patients with both high CEA levels and positive citology in peritoneal washings taken at the beginning of surgery (Kanellos *et al*, 2003, 2006b). Several patients in the present series were referred to our unit from a neighbouring hospital with preoperative CEA often not technically comparable or even not assessed. However, we know that apart from cases with documented distant metastases (stage IV), some patients have CEA levels higher than normal after putatively radical operations, without any instrumental macroscopic evidence of persisting local residual disease or distant spread. Since the half-life of CEA serum level has been calculated to be a few days – from 3.0 (Rapellino *et al*, 1994) to 4.3 (Ito *et al*, 2002) or 6.2 (Choi and Min, 1997) – our evaluation at a mean interval of 42 days after surgery – and rarely after less than 4 weeks – seems able to reliably reflect the presence of occult spread of the disease. In such cases, the clinical problem is whether to plan adjuvant therapy according to the TNM stage only or whether to consider also the altered marker value as an important worsening factor.

The clinical importance of CEA is clear at whatever time it is assessed along the clinical course of patients with colorectal cancer, but the demonstration of it having a prognostic role also postoperatively would be interesting for deciding treatment strategies. Besides providing a serologic indication of how radical the resection has been, in addition to the mandatory microscopic inspections by the histopathologists – it could offer further information to guide the choice of subsequent treatment. This decisional step is becoming ever more critical given the large number of available adjuvant chemotherapy regimens, with selective indications and different intensities and toxicities. Since the ultimate fate of the patients with colorectal cancer is related to three main factors (initial tumour stage, radicality of the surgical resection and effectiveness of adjuvant therapy – when necessary), the postoperative evaluation of the CEA level (after 5–6 weeks), in that it allows a reliable check of the last two factors, can be at least as important as the preoperative one. Thus, we fully agree with the proposal of the American Joint Committee on Cancer (Compton *et al*, 2000), which recommends the inclusion of CEA into the TNM classification (with the following suggested notations for any stage: CX, CEA not assessed; C0, CEA not elevated; and C1, CEA elevated). We only wonder whether the measurements of CEA for such a categorisation should be more properly performed at a suitable interval after surgery (we suggest 5–6 weeks) – instead of or in addition to before surgery.

In conclusion, our study of the relative survival in a population of patients who had undergone radical resection of colorectal cancer fully confirms the adequacy of the individual clinicopathological factors, which are the basis of the current Dukes', modified Astler–Coller and TNM staging classifications. The results presented offer a possible explanation for the conflicting reports on the prognostic role of age and, finally, show the true prognostic value – strictly related to cancer – of the CEA level, with particular emphasis on its postoperative assessment.

## Figures and Tables

**Table 1:** Main clinical and pathological characteristics of the 506 patients of the study (values between parentheses, ranges between brackets)

Sex (male: 292; female: 214)	Age (mean=64.3 years [29–83])
Days from surgery to chemotherapy:^a^ 42±14	Tumour site (colon: 324; rectum: 182)
Tumour shape (ulcerated: 265; nonulcerated: 241)	Tumour size (largest ∅: 4.8±2.4 cm [0.5–18]
Depth of involvement (T_1_: 11; T_2_: 31; T_3_: 400; T_4_: 64)	Histological grading (G_1_: 5; G_2_: 421; G_3_: 69; G_4_: 11)
Angioinvasion (yes: 47; no: 459)	Lymphoinvasion (yes: 151; no: 355)
Neuroinvasion (yes: 18; no: 488)	Lymphocyte infiltration (yes: 358; no: 148)
Regional node involvement (yes: 263; no: 243)	Distant node involvement (yes: 22; not: 484)
Presence of liver metastases (yes: 74; no: 432)	Number of liver metastases (median=1 [1–14]
Extrahepatic metastases (yes: 43; no: 463)	Haemoglobin (g per 100 ml: 12.1±1.7 [7.6–16.0])
Serum albumin (g per 100 ml: 4.02±0.52 [2.60–5.63])	Serum CEA (ng ml^−1^: mean=4.3 [0.1–6.000])
Serum CA19-9 (U ml^−1^: mean=9.1 [0.1–11.000])	Dukes' stages (A: 22; B: 183; C: 176; D: 125)
MAC stages (A: 16; B: 189; C: 176; D: 125)	TNM stages (TI: 16; TII: 189; TIII: 176; TIV: 125)

a In patients who did not undergo chemotherapy, the interval was calculated from surgery to the first clinical follow-up evaluation.

**Table 2:** Results of univariate analysis for overall survival and relative survival (qualitative data categorised as in Table 1)

	**Overall survival**		**Relative survival**	
**Variables**	***χ* ^2^**	***P*-value**	***χ* ^2^**	***P*-value**
Sex	0.181	0.6705	21.408	<0.0001
Age	18.379	<0.0001	23.354	<0.0001
Tumour site	0.991	0.3194	0.780	0.3772
Tumour shape	2.680	0.1016	2.179	0.1399
Tumour size	0.843	0.3587	0.209	0.6477
Depth of involvement	22.834	<0.0001	14.782	0.0001
Histological grading	4.053	0.0441	4.885	0.0271
Angioinvasion	1.660	0.1977	1.582	0.2084
Lymphoinvasion	15.258	<0.0001	16.018	<0.0001
Neuroinvasion	3.725	0.0536	4.865	0.0274
Lymphocyte infiltration	0.185	0.6675	0.002	0.9912
Regional node involvement	108.770	<0.0001	82.777	<0.0001
Distant node involvement	4.394	0.0361	5.348	0.0207
Presence of liver metastases	73.541	<0.0001	63.179	<0.0001
Number of liver metastases	73.754	<0.0001	54.246	<0.0001
Extrahepatic metastases	35.407	<0.0001	27.350	<0.0001
Haemoglobin	1.261	0.2614	0.009	0.9261
Serum albumin	0.825	0.3636	0.734	0.3915
Serum CEA	10.965	0.0009	14.263	0.0002
Serum CA19-9	12.808	0.0003	23.058	<0.0001
Dukes' stages	121.595	<0.0001	96.964	<0.0001
MAC stages	111.716	<0.0001	87.425	<0.0001
TNM stages	120.418	<0.0001	95.893	<0.0001

CA19-9=carbohydrate antigen 19-9; CEA=carcinoembryonic antigen.

**Table 3:** Multivariate analysis for overall survival and relative survival

	**Overall survival**		**Relative survival**	
**Variables**	**Coefficient**	***P*-value**	**Coefficient**	***P*-value**
Presence of liver metastases	1.209	<0.0001	1.072	<0.0001
Age	0.039	<0.0001	−0.054	<0.0001
No. of involved regional nodes	0.115	<0.0001	0.089	<0.0001
Depth of involvement	0.351	0.0313	0.373	0.0092
Postoperative CEA	0.001	0.0925	0.001	0.0321

CEA=carcinoembryonic antigen.

Final results after stepwise selection of the best clinical parameters.

## References

[bib1] American Joint Committee on Cancer (2002) Cancer Staging Manual, 6th edn, pp 113–119. Springer-Verlag: New York

[bib2] Andre T, Louvet C, Maindrault-Goebel F, Couteau C, Mabro M, Lotz JP, Gilles-Amar V, Krulik M, Carola E, Izrael V, De Gramont A (1999) CPT-11 (irinotecan) addition to bimonthly, high-dose leucovorin and bolus and continuous-infusion 5-fluorouracil (FOLFIRI) for pretreated and metastatic colorectal cancer. GERCOR. Eur J Cancer 35: 1343–1347 10658525 10.1016/s0959-8049(99)00150-1

[bib3] Armitage P, Berry G (1987) Statistical Methods in Medical Research, 2nd edn, Oxford: Blackwell Scientific Publication, pp 421–437

[bib4] Astler VB, Coller FA (1954) The prognostic significance of direct extension of carcinoma of the colon and the rectum. Ann Surg 139: 707–711 10.1097/00000658-195406000-00015 PMC1609522 13159135

[bib5] Cai SR, Zheng S, Zhang SZ (2005) Multivariate analysis of prognostic factors in colorectal cancer. Zhonghua Zhong Liu Za Zhi 27: 483–485 16188146

[bib6] Cerottini JP, Caplin S, Pampallona S, Givel JC (1999) Prognostic factors in colorectal cancer. Oncol Rep 6: 409–414 10023012 10.3892/or.6.2.409

[bib7] Chen CC, Yang SH, Lin JK, Lin TC, Chen WS, Jiang JK, Wang LW (2005) Is it reasonable to add preoperative level of CEA and CA19-9 to staging for colorectal cancer? J Surg Res 124: 169–174 15820244 10.1016/j.jss.2004.08.013

[bib8] Choi JS, Min JS (1997) Significance of postoperative serum level of carcinoembryonic antigen (CEA) and actual half life of CEA in colorectal cancer patients. Yonsei Med J 38: 1–7 9100477 10.3349/ymj.1997.38.1.1

[bib9] Chung YE, Eu KW, Machin D, Ho JM, Leong AF, Ho YU, Seow-Choen F (1998) Young age is not a poor prognostic factor in colorectal cancer. Br J Surg 85: 1255–1259 9752871 10.1046/j.1365-2168.1998.00805.x

[bib10] Compton C, Fenoglio-Praser CM, Peltigrew N, Peter Fielding L (2000) American Joint Committee on Cancer Prognostic Factors Consensus Conference. Colorectal Cancer Group. Cancer 88: 1739–1757 10738234 10.1002/(sici)1097-0142(20000401)88:7<1739::aid-cncr30>3.0.co;2-t

[bib11] Cox DR (1972) Regression models and life tables. J R Stat Soc 34: 187–220

[bib12] Crawford NPS, Colliver DW, Galandiuk S (2003) Tumor markers and colorectal cancer: utility in management. J Surg Oncol 84: 239–248 14756436 10.1002/jso.10325

[bib13] Crocetti E, Barchielli A, Buiatti E, Castiglione G, Zappa M (1996) Colorectal cancer survival – population-based rates in the province of florence. Eur J Cancer Prev 5: 189–195 8818608 10.1097/00008469-199606000-00007

[bib14] D'Eredita G, Serio G, Neri Y, Polizzi RA, Barberio G, Losacco T (1996) A survival regression analysis of prognostic factors in colorectal cancer. Aust NZ J Surg 66: 445–451 10.1111/j.1445-2197.1996.tb00780.x 8678873

[bib15] De Gramont A, Bosset JF, Milan C, Rougier P, Bouche O, Etienne PL, Morvan F, Louvet C, Guillot T, François E, Bedenne L (1997) Randomized trial comparing monthly low-dose leucovorin and fluorouracil bolus with bimonthly high-dose leucovorin and fluorouracil bolus plus continuous infusion for advanced colorectal cancer: a French Intergroup study. J Clin Oncol 15: 808–815 9053508 10.1200/JCO.1997.15.2.808

[bib16] De Gramont A, Figer A, Seymour M, Homerin M, Hmissi A, Cassidy J, Boni C, Cortes-Funes H, Cervantes A, Freyyer G, Papamichael D, Le Bali N (2000) Leucovorin and fluorouracil with or without oxaliplatin as first-line treatment in advanced colorectal cancer. J Clin Oncol 16: 2938–2947 10.1200/JCO.2000.18.16.2938 10944126

[bib17] Dukes CE (1940) Cancer of the rectum: an analysis of 1000 cases. J Pathol Bacteriol 50: 527–539

[bib18] Fietkau R, Zetti H, Klocking S, Kundt G (2004) Incidence, therapy and prognosis of colorectal cancer in different age groups. A population-based cohort study of the Rostock Cancer Registry. Strahlenther Onkol 180: 478–487 15292968 10.1007/s00066-004-1260-z

[bib19] Frank RE, Saclarides TJ, Leurgans S, Speziale NJ, Drab EA, Rubin DB (1995) Tumor angiogenesis as a predictor of recurrence and survival in patients with node-negative colon cancer. Ann Surg 222: 695–699 8526575 10.1097/00000658-199512000-00002 PMC1235017

[bib20] Gasser A, Isaak B, Maibach R, Ruchti C, Wagner HE, Nothiger E (1992) Staging and prognosis of colorectal carcinoma. Schweiz Med Wochenschr 122: 1356–1362 1411393

[bib21] Herrera MA, Chu TM, Holyoke ED (1976) Carcinoembryonic antigen (CEA) as a prognostic and monitoring test in clinically complete resection of colorectal carcinoma. Ann Surg 183: 5–9 1247300 10.1097/00000658-197601000-00002 PMC1344173

[bib22] Heys SD, Walker LG, Deehan DJ, Eremin OE (1998) Serum albumin: a prognostic indicator in patients with colorectal cancer. J R Coll Surg Edinb 43: 163–168 9654876

[bib23] Ito K, Hibi K, Ando H, Hidemura K, Yamazaki T, Akiyama S, Nakao A (2002) Usefulness of analytical CEA doubling time and half-life time for overlooked synchronous metastases in colorectal carcinoma. Jpn J Clin Oncol 32: 54–58 11948229 10.1093/jjco/hyf011

[bib24] Janssen-Heijnen ML, Houterman S, Lemmens VE, Louwman MW, Maas HA, Coeberg JW (2005) Prognostic impact of increasing age and co-morbidity in cancer patients: a population-based approach. Clin Rev Oncol Hematol 55: 231–240 10.1016/j.critrevonc.2005.04.008 15979890

[bib25] Kanellos I, Demetriades H, Zintzaras E, Mandrali A, Mantzoros I, Betsis D (2003) Incidence and prognostic value of positive peritoneal cytology in colorectal cancer. Dis Colon Rectum 46: 535–539 12682550 10.1007/s10350-004-6595-0

[bib26] Kanellos I, Zacharakis E, Demetriades H, Christoforidis E, Kanellos D, Pramateftakis M-G, Betsis D (2006a) Value of carcinoembryonic antigen assay in predicting hepatic metastases, local recurrence, and survival after curative resection of colorectal cancer. Surg Today 36: 879–884 16998681 10.1007/s00595-006-3272-z

[bib27] Kanellos I, Zacharakis E, Kanellos D, Pramateftakis M-G, Betsis D (2006b) Prognostic significance of CEA levels and positive cytology in peritonela washings in patients with colorectal cancer. Colorectal Dis 8: 436–440 16684089 10.1111/j.1463-1318.2006.00991.x

[bib28] Kanellos I, Zacharakis E, Kanellos D, Pramateftakis M-G, Tsahalis T, Altsitsiadis E, Betsis D (2006c) Prognostic significance of CEA levels and detection of CEA mRNA in draining venous blood in patients with colorectal cancer. J Surg Oncol 94: 3–8 16788936 10.1002/jso.20549

[bib29] Kaplan EL, Meier P (1958) Nonparametric estimation from incomplete observations. J Am Stat Assoc 53: 457–481

[bib30] Korenaga D, Ueo H, Mochida K, Kusumoto T, Baba H, Tamura S, Moriguchi S, Sugimachi K (1991) Prognostic factors in Japanese patients with colorectal cancer: the significance of large bowel obstruction – univariate and multivariate analyses. J Surg Oncol 47: 188–192 2072703 10.1002/jso.2930470310

[bib31] Lagautriere F, Valvano L, Chaazl M, Benchimol D, Bernard JL, Bourgeon A, Richelme H (1998) Prognostic factors in colorectal cancer. Ann Ital Chir 69: 491–497 9835125

[bib32] Latkauskas T, Rudinskaite G, Kurtinaitis J, Janciauskiene R, Tamelis A, Saladzinskas Z, Pawalkis D (2005) The impact of age on post-operative outcomes of colorectal cancer patients undergoing surgical treatment. BMC Cancer 5: 153–158 16324216 10.1186/1471-2407-5-153 PMC1318482

[bib33] Louhimo J, Carpelan-Holmstrom M, Alfthan H, Stenman UH, Jarvinen HJ, Haglund C (2002) Serum HCG beta, CA72-4 and CEA are independent prognostic factors in colorectal cancer. Int J Cancer 101: 545–548 12237895 10.1002/ijc.90009

[bib34] Machover D, Schwarzenberg L, Goldschmidt E, Tourani JM, Michalski B, Hayat M, Dorval T, Misset JL, Jasmin C, Maral R, Mathe G (1982) Treatment of advanced colorectal and gastric adenocarcinoma with 5-FU combined with high-dose folinic acid: a pilot study. Cancer Treat Rep 66: 1803–1807 6982099

[bib35] Massacesi C, Pistilli B, Valeri M, Lippe P, Rocchi MB, Cellerino R, Piga A (2002) Predictors of short-term survival and progression to chemotherapy in patients with advanced colorectal cancer treated with 5-fluorouracil-based regimens. Am J Clin Oncol 25: 140–148 11943891 10.1097/00000421-200204000-00008

[bib36] Matthes M, Lucas A, Boland R, Hirth V, Odenheimer G, Wieland D, Williams H, Eleazer GP (2004) Use of a questionnaire to screen for frailty in the elderly: an exploratory study. Aging Clin Exp Res 16: 34–40 15132289 10.1007/BF03324529

[bib37] Menin C, Scaini MC, De Salvo GL, Biscuola M, Quaggio M, Esposito G, Belluco C, Montagna M, Agata S, D'Andrea E, Nitti D, Amadori A (2006) Association between MDM2-SNP309 and age at colorectal cancer diagnosis according to p53 mutation status. J Natl Cancer Inst 98: 285–288 16478747 10.1093/jnci/djj054

[bib38] Mitry E, Douillard J-Y, Van Cutsem E, Cunningham D, Magherini E, Mery-Mignard D, Awad L, Rougier P (2004) Predictive factors of survival in patients with advanced colorectal cancer: an individual data analysis of 602 patients included in irinitecan phase III trials. Ann Oncol 15: 1013–1017 15205193 10.1093/annonc/mdh267

[bib39] Munemoto Y, Iida Y, Ohata K, Saito H, Fujisawa K, Kasahara Y, Mitsui T, Asada Y, Miura S (2004) Significance of postoperative adjuvant immunochemotherapy after curative resection of colorectal cancers: identification of responders incorporating the age factor. Oncol Rep 11: 623–635 14767513

[bib40] Newland RC, Dent OF, Lyttle MN, Chapuls PH, Bokey EL (1994) Pathologic determinants of survival associated with colorectal cancer with lymph node metastases. A multivariate analysis of 579 patients. Cancer 78: 403–408 10.1002/1097-0142(19940415)73:8<2076::aid-cncr2820730811>3.0.co;2-6 8156513

[bib41] Payne JE, Meyer HJ (1997) Independently predictive prognostic variables after resection for colorectal carcinoma. Aust NZ J Surg 67: 849–853 10.1111/j.1445-2197.1997.tb07610.x 9451339

[bib42] Peto R, Pike MC, Armitage P, Breslow NE, Cox DR, Howard SV, Mantel N, McPherson K, Peto J, Smith PG (1977) Design and analysis of randomized clinical trials requiring prolonged observation of each patient. II. Analysis and examples. Br J Cancer 35: 1–39 831755 10.1038/bjc.1977.1 PMC2025310

[bib43] Ponz de Leon M, Sant M, Micheli A, Sacchetti C, Di Gregorio C, Fante R, Zanghieri G, Melotti G, Gatta G (1992) Clinical and pathological prognostic indicators in colorectal cancer. Cancer 69: 626–635 1730115 10.1002/1097-0142(19920201)69:3<626::aid-cncr2820690305>3.0.co;2-#

[bib44] Rapellino M, Piantino P, Pecchio F, Ruffini E, Cavallo A, Scappaticci E, Baldi S, Ciocia E, Pivetti S (1994) Disappearance curves of tumor markers after radical surgery. Int J Biol Markers 9: 33–37 8051433 10.1177/172460089400900107

[bib45] Repetto L, Comandini D, Mammoliti S (2001) Life expectancy, comorbidity and quality of life: the treatment equation in the older cancer patients. Crit Rev Oncol Hematol 37: 147–152 11166588 10.1016/s1040-8428(00)00104-9

[bib46] Takahashi Y, Tucker SI, Kitadal Y, Koura AN, Bucana DC, Cleary KR, Ellis LM (1997) Vessel counts and expression of vascular endothelial growth factor as prognostic factors in node-negative colon cancer. Arch Surg 132: 541–546 9161399 10.1001/archsurg.1997.01430290087018

[bib47] Tominaga T, Sakabe T, Koyama Y, Hamano K, Yasutomi M, Takahashi T, Kodaira S, Kato T, Ogawa N (1997) Prognostic factors for patients with colon or rectal carcinoma treated with resection only. Five-year follow-up report. Cancer 78: 403–408 10.1002/(SICI)1097-0142(19960801)78:3<403::AID-CNCR4>3.0.CO;2-K 8697383

[bib48] Wanebo HJ, Rao B, Pinsky CM, Hoffman RG, Stearns M, Schwartz MK, Oettgen HF (1978) Preoperative carcinoembryonic antigen level as a prognostic indicator in colorectal cancer. N Engl J Med 299: 448–451 683276 10.1056/NEJM197808312990904

[bib49] Wang WS, Chen PM, Chiou TJ, Liu JH, Fan FS, Lin TC, Jiang JK, Yang SH, Chen PM (2000) Factors predictive of survival in patients with node-positive colorectal cancer. Hepatogastroenterology 47: 1590–1594 11149009

[bib50] Wolters U, Stutzer H, Keller HW, Schroder U, Pichlmaier H (1996) Colorectal cancer – a multivariate analysis of prognostic factors. Eur J Surg Oncol 22: 592–597 9005146 10.1016/s0748-7983(96)92320-3

[bib51] Yuste AL, Aparicio J, Segura A, Lopez-Tendero P, Girones R, Perez-Fidalgo JA, Diaz R, Calderero V (2003) Analysis of clinical prognostic factors for survival and time to progression in patients with metastatic colorectal cancer treated with 5-fluoruracil-based chemotherapy. Clin Colorectal Cancer 2: 231–234 12620142 10.3816/CCC.2003.n.004

